# Extraction frequent patterns in trauma dataset based on automatic generation of minimum support and feature weighting

**DOI:** 10.1186/s12874-024-02154-0

**Published:** 2024-02-16

**Authors:** Zahra Kohzadi, Ali Mohammad Nickfarjam, Leila Shokrizadeh Arani, Zeinab Kohzadi, Mehrdad Mahdian

**Affiliations:** 1 Health Information Management Research Center, Kashan University of Medical Sciences, Kashan, Iran; 2 Department of Health Information Management and Technology, Allied Medical Sciences Faculty, Kashan University of Medical Sciences, Kashan, Iran; 3 Medical Informatics Department, School of Allied Medical Sciences Shahid, Beheshti University of Medical Sciences, Tehran, Iran; 4 Trauma Research Center, Kashan University of Medical Sciences, Kashan, Iran

**Keywords:** Association rule mining, Apriori algorithm, Automatic minimum support, Trauma registry

## Abstract

**Purpose:**

Data mining has been used to help discover Frequent patterns in health data. it is widely used to diagnose and prevent various diseases and to obtain the causes and factors affecting diseases. Therefore, the aim of the present study is to discover frequent patterns in the data of the Kashan Trauma Registry based on a new method.

**Methods:**

We utilized real data from the Kashan Trauma Registry. After pre-processing, frequent patterns and rules were extracted based on the classical Apriori algorithm and the new method. The new method based on the weight of variables and the harmonic mean was presented for the automatic calculation of minimum support with the Python.

**Results:**

The results showed that the minimum support generation based on the weighting features is done dynamically and level by level, while in the classic Apriori algorithm considering that only one value is considered for the minimum support manually by the user. Also, the performance of the new method was better compared to the classical Apriori method based on the amount of memory consumption, execution time, the number of frequent patterns found and the generated rules.

**Conclusions:**

This study found that manually determining the minimal support increases execution time and memory usage, which is not cost-effective, especially when the user does not know the dataset's content. In trauma registries and massive healthcare datasets, its ability to uncover common item groups and association rules provides valuable insights. Also, based on the patterns produced in the trauma data, the care of the elderly by their families, education to the general public about encountering patients who have an accident and how to transport them to the hospital, education to motorcyclists to observe safety points in Recommended when using a motorcycle.

## Introduction

Trauma poses a significant global health challenge, exerting a profound impact on individuals worldwide and standing as the primary cause of mortality among individuals under the age of 45 [1]. Notably, over half of fatalities occur within minutes of sustaining an injury, often beyond the reach of immediate medical attention despite the presence of well-established trauma systems [2]. Findings from Study Mock et al [3] revealed a significant correlation between the economic status of a country and mortality rates attributed to trauma. The results indicated that in Ghana, an injured patient faces nearly double the risk of mortality compared to a patient with identical injuries in the United States.

The introduction of trauma care systems in high-income countries has yielded remarkable reductions in both mortality and disability rates. It is estimated that by enhancing trauma care systems on a global scale, approximately one-third of deaths resulting from injuries could be prevented [1]. Trauma registries were initially conceived as a tool for enhancing the quality of care provided, offering a wealth of valuable clinical information. Typically, these registries encompass key components such as the Abbreviated Injury Scale (AIS), details on prevention measures, pre-hospital care, and post-discharge care [4]. Typically, trauma registries encompass a wide range of data, covering patient demographics, injury circumstances, pre-hospital care and transportation details, emergency department interventions, in-hospital treatments, anatomical injury descriptions, physiological measurements, complications, outcomes, and patient dispositions. Moreover, these registries are progressively incorporating information on pre-existing medical conditions, which are recognized as significant determinants of outcomes independent of age and injury severity [5].

The size of data is consistently expanding, and the demand to comprehend extensive and information-rich datasets has risen across various domains such as technology, business, and science. In today's competitive world, where large volumes of data are prevalent, the ability to extract valuable insights from these datasets and utilize that knowledge has become increasingly crucial. The practice of employing computer-based information systems, along with innovative techniques, to uncover knowledge from data is known as data mining [6]. Data mining plays a crucial role in the healthcare sector by enabling knowledge discovery and pattern identification to facilitate decision-making processes. It stands as a rapidly advancing field focused on extracting valuable and meaningful insights from extensive datasets. Within healthcare, data mining employs analytical methodologies to identify vital information that supports decision-making processes. Its importance spans various areas, including disease detection, prevention, and management, fraud detection in health insurance, reduction of medical care costs, and the development of effective healthcare policies. Additionally, data mining aids researchers in creating recommendation systems, patient health profiles, and overall contributes to improved diagnosis and treatment through the storage and analysis of voluminous healthcare data using database systems [7].

Data mining, also known as knowledge discovery in databases (KDD), involves the collection and analysis of historical data to identify patterns, relationships, or regularities within large datasets. The results of data mining can provide valuable insights for making informed decisions in the future. With the evolution of KDD, the use of pattern recognition has become integrated into data mining, leading to a decrease in the reliance on standalone pattern recognition techniques [8].

The data mining industry is actively conducting research in the association rules mining field [9, 10]. In recent times, various algorithms have been suggested for extracting discovered patterns by mining association rules [11]. The Apriori algorithm is a widely-used algorithm for mining association rules in transaction databases. It was the first such technique developed and remains one of the most popular methods for identifying frequent itemset and interesting associations [12]. The Apriori algorithm is a classic and pioneering association rule mining algorithm that uses a layer-by-layer iterative search approach to discover relationships between item sets in a database and generate rules [13].

In the field of health, many studies have been done using Apriori, and association rules mining such as heart diseases [14, 15], Alzheimer’s disease diagnosis [16], Cancer Diagnoses [17], Diabetes Medical History [18], Predicting the Risk of Diabetes Mellitus [19], chronic inflammatory diseases [20].

Since trauma registries produce vast amounts of diverse and intricate data, using association rules mining for exploratory analysis can help uncover novel, interesting, and obscure patterns. Several of these studies are listed below.

According to the research of Fagerlind et al [21], the Swedish Traffic Accident Data Acquisition was utilized to analyze crash circumstances reported by the police and injury information gathered from hospitals during the years 2011 to 2017. By applying the Apriori algorithm, statistical associations between injuries (IBIP) were identified through the analysis of injury data. Out of the 48,544 individuals analyzed, 36,480 (75.1%) had a single recorded injury category, while 12,064 (24.9%) had multiple injuries. The analysis using data mining techniques revealed 77 IBIPs among the multiply injured individuals, and out of these, 16 were linked to only one type of road user.

The study of Karajizadeh et al [22] is classified as a cohort study, which involved analyzing 549 trauma patients with nosocomial infections who were admitted to Shiraz trauma hospital between 2017 and 2018. The study collected data on various factors such as sex, age, mechanism of injury, body region injured, injury severity score, length of stay, type of intervention, infection day after admission, microorganism cause of infections, and outcomes. Knowledge was extracted from the dataset using association rule mining techniques, and the IBM SPSS Modeler data mining software version 18.0 was utilized as a tool for data mining of the trauma patients with hospital queried infections database. Their results showed that the following factors were found to be associated with in-hospital mortality at a confidence level of over 71%: age over 65, surgical site infections on the skin, bloodstream infections, injuries caused by car accidents, invasive tracheal intubation procedures, injury severity scores above 16, and multiple injuries.

The objective of the study by Aekwarangkoon et al [23] was to utilize association rule mining to identify related patterns, and subsequently, to develop a prediction model utilizing ensemble learning techniques to predict high levels of depression and suicide rates among primary school students attending extended opportunity schools in rural Thailand, where incidents of trauma are prevalent. The results of the experiment indicated that the crucial feature items identified in this study surpassed all other previously used items in predicting depression and suicide. Furthermore, the approach proposed in this research can serve as an initial screening process for identifying individuals at risk of depression and suicide.

The Research by Finley et al [24] was conducted with the aim of investigating the potential effects of traumatic brain injury on the capacity to classify and remember visual signals from a subjective perspective. They use the Association Rule Modelling method to measure Subjective organization and examine whether the complexity of Association Rule Modelling -generated rules predicts symbol recall.

In the study of Sarıyer et al [25], the real-life medical data obtained from an emergency department was analyzed using association rule mining to uncover hidden patterns and relationships between diagnostic test requirements and diagnoses. The diagnoses were classified into 21 categories according to the International Classification of Diseases, while the laboratory tests were grouped into four main categories. The study demonstrated that identifying the correlation between a patient's diagnosis and their required diagnostic tests can enhance decision-making and optimize resource utilization in emergency departments. Furthermore, association rules can aid physicians in treating patients effectively.

But in all these studies, classical Apriori algorithm have been used to extract frequent rules and patterns.

Association rule mining comprises a two-step procedure: (i) identifying frequent itemsets within the dataset, and (ii) deriving inferences from these identified itemsets. The identification of frequent itemsets is acknowledged as the computationally most challenging task in this process and has been demonstrated to be NP-Complete [26].

The essential component rendering association-rule mining feasible is the minimum support threshold, referred to as minsup. Its primary function is to prune the search space and constrain the number of generated rules. Nonetheless, relying solely on a single minsup presupposes that all items in the database share the same nature or exhibit comparable frequencies, which may not accurately represent real-life applications [27, 28].

Establishing an inaccurate minimum support (min_sup) threshold can lead to two significant issues: (i) the algorithm's failure to identify correlated patterns, and (ii) a potentially more serious problem, wherein the algorithm may produce misleading patterns that do not genuinely exist [29].

This is particularly probable when an analyst lacks a comprehensive understanding of the significance of an input parameter in the data mining process or fails to choose optimal parameter values. Such oversights can result in the algorithm's failure to identify highly correlated patterns [30].

The primary challenge with the Apriori algorithm is picking the support and confidence thresholds. Apriori finds the most common candidate itemset by making every possible candidate itemset that meets a minimum support set by the user. This decision affects how many association rules there are and what kind of association rules they are. In practical applications, users cannot discover a suitable minimum support value immediately and must constantly tune it. To accomplish this, every time a user modifies an item's minimum support, they must again scan the database and repeat the mining algorithm. Also, not all elements in a itemset act in the same way; some appear regularly and frequently, while others appear occasionally and rarely [31]. Also, if the threshold value is too small, it will generate many useless rules, and if it is too large, it may cause useful information to be deleted [32].

It is extremely time-consuming and expensive. Thus, it is appealing to think of the possibility of designing an algorithm for automatically generating minimum supports. As a result, the minimum support threshold should be adjusted based on the element set's various levels. The works of others are mentioned in the section on related works.

On the other hand, the advantage of using the weight of variables in extracting frequent patterns is stated in various studies [33, 34]. According to these studies, an item may exist many times in the database, but it is not very important, as a result, the importance (their weight) of the variables can be effective in extracting frequent patterns.

Therefore, the aim of this study is to extract frequent patterns in the data of Kashan Trauma Center using an improved algorithm based on the creation of automatic minimum support and feature weights.

At the end of this study, the following questions will be answered:

What is the new method to automatically calculate the minimum support in the Apriori algorithm?

What is the effect of weighting variables to produce frequent patterns?

What is the impact of the new method on algorithm execution time, memory consumption, the number of frequent patterns, and the quality of generated rules?

The other sections of the paper are as follows: in Section " Methodology", the methodology is explained, including the description of the dataset, the selection of the algorithm, evaluation, and Implemented framework. In Sections " Experimental Results", the findings of the experiments are presented. Finally, in Section " Discussion", the conclusion is stated.

### Related works

Table 1 presents a comparative assessment of prior studies, delving into aspects such as their objectives, use of real datasets, introduction of implementation platforms, evaluation indicators, and the delineation of respective advantages and limitations. This comparative overview provides a comprehensive insight into the diverse approaches embraced in the field, fostering a nuanced understanding of the strengths and weaknesses inherent in each study.

**Table 1:** Related work

**Study**	**Proposed Method**	**Real DataSet**	**How to change min support**	**Framework**	**Evaluation**	**Advantage**	**Constrain**
Bing Liu [27]	In this study, the authors expand upon the current association rule model, enabling users to define multiple minimum supports to capture diverse natures and frequencies of items. More specifically, users have the capability to designate distinct minimum item support levels for each individual item.	√	Multiple min sup	----	Number of large item set Number of Candidate item set	This model allows for the discovery of rare item rules without generating an excessive number of irrelevant rules associated with frequent items. The experimental and practical demonstrations underscore the effectiveness of this novel model.	In the past, users were only required to adjust a single minimum support (MS) threshold, but now they find themselves needing to fine-tune multiple MS thresholds.
Ya-Han Hu [35]	In this research, the emphasis is placed on the upkeep of the MIS-tree. This ensures that following any adjustments made to the item supports, the MIS-tree remains in a correct and accurate state without necessitating a rescan of the database.	√	Multiple min sup	----	Runtime	They observe that their CFP-growth algorithm outperforms the MSapriori algorithm by approximately an order of magnitude across all datasets. The development of their efficient algorithm for mining frequent patterns with multiple minimum support values is a notable achievement.	They address a limitation in the MSapriori algorithm, which necessitates a post-processing phase for the generation of association rules. Additionally, they introduce an efficient maintenance algorithm for updating the MIS-tree when the user adjusts the MIS values of items. However, the process of determining the minimum support still involves user intervention.
R. Uday [36]	In this study, the authors investigated the concept of "item-to-pattern difference" and expanded its application to the minimum constraint model. This extension allows the model to effectively prune patterns during the process of mining rare association rules.	√	Multiple min sup	----	Number of Frequent patterns	The findings indicate that, in comparison to both the single minimum support (minsup) model and the minimum constraint model, the proposed model effectively eliminates a higher number of uninteresting rules during the mining of rare association rules.	It shares the same issues as the minimum support (MS) model. There is still parameter setting by the user
Salam [29]	They introduce an innovative approach to efficiently retrieve the top few maximal frequent patterns in order of significance, eliminating the need for the minimum support parameter.	----	Automatic minsup	----	Runtime	Their approach involves a single pass through the database and the generation of length two itemsets. The association ratio graph is introduced as a compact structure that encapsulates concise information and is constructed in time proportional to the square of the database size.	They only need to designate a parameter that is more easily understandable for humans, namely the desired number of itemsets, denoted as "k."
Kuo [37]	This research aims to introduce an innovative algorithm for association rule mining with the goal of enhancing computational efficiency and automating the identification of appropriate threshold values. The proposed method employs the particle swarm optimization algorithm, which initially seeks the optimal fitness value for each particle.	√	Automatic minsup	Borland C++ Builder 6	Runtime Number of frequently generated patterns	This research has illustrated that the utilization of the PSO algorithm enables the rapid and objective determination of these two parameters. As a result, it enhances the mining performance for large databases, as evidenced by its application to the FoodMart2000 database.	varying product items may carry distinct levels of significance. Exploring a weighted PSO mining algorithm could offer additional practical approaches for industries.
Azzeddine Dahbi [32]	In this paper, the authors introduce two primary contributions. The initial contribution involves the automated computation of the minimum support (minsup) based on each dataset, rather than relying on a predetermined constant value set by users. The second contribution of their proposed method is the dynamic update of this minsup at each level, achieved by utilizing the means of support for all itemsets with a single item.	√	Automatic minsup	----	Number of Association Rule Time-consuming Quality of the Extracted Rules	The primary benefit of the proposed approach lies in the automatic determination of support at multiple levels. It yields results that encompass the desired rules with maximum interestingness in a short timeframe. The number of rules generated by the proposed algorithm is considerably lower when compared to the APRIORI Algorithm.	It employs the Mean for the automated calculation of the minimum support, a method that is susceptible to outlier data. This sensitivity may impact the algorithm's overall performance.
Azzeddine Dahbi [33]	The primary innovation in their paper lies in the automatic calculation of the minsup threshold, which is tailored to each dataset, departing from the conventional approach of using a user-predefined constant value. To accomplish this objective, the authors employ a set of statistical measures, encompassing central tendency measures like mean, mode, and median, as well as dispersion measures such as range, standard deviation, quartile 1, and quartile 3.	√	Automatic minsup	----	Number of Association Rule Time-consuming Quality of the Extracted Rules	They achieve results that encompass the desired rules with maximum interestingness in a short period. The proposed algorithm generates significantly fewer rules in comparison to the Apriori algorithm.	In spite of advancements in enhancing min support calculation compared to the preceding method, the impact of variable importance has been overlooked.

The examination of each study involves a critical assessment of its purpose, revealing the specific goals and objectives pursued by the researchers. The utilization of real datasets serves as a significant criterion, indicating the practical applicability and relevance of the proposed methods. Additionally, the implementation platform signifies the technology or programming language employed in the study, offering insights into the technical aspects of the research. Evaluation indicators are crucial for gauging the performance of proposed methods, encompassing metrics such as RAM memory consumption, hard disk space utilization, algorithm execution time, the count of frequently generated patterns, the number of generated rules, and the quality of these rules. A comprehensive analysis of these indicators provides valuable insights into the effectiveness and efficiency of each study. Moreover, it is essential to take into account both the strengths and limitations of each study. Recognizing the strengths aids in identifying innovative aspects and potential contributions, while being cognizant of limitations is crucial for placing the findings in context and pinpointing areas for improvement.

In past studies, despite efforts to solve the problem of minimum single support as well as generate association rules based on the weight of variables, However, the weighting of variables and the automation of the minimum support, eliminating the need for user intervention, have not been implemented simultaneously.

Therefore, the current study endeavors to incorporate both variable weighting and automated minimum support calculation.

## Methodology

In this study, our goal is to improvement the calculation of min support and discover frequent patterns in trauma data by incorporating variable weights.

### Dataset

For this research, the data from March 2018 to February 2019 the Kashan Trauma Centre was utilized.

The data pre-processing involved multiple steps:

- Noisy and outlier data were removed.
- Numerical variables were imputed using the mean, while categorical variables were imputed using the mode.
- Normalize Min-Max was then utilized to normalize the data.
- Lastly, one hot encoding was employed to discretize the data.

One-hot encoding is a machine learning approach for representing categorical variables as numbers so that algorithms can process them. This is done by building a binary vector with one element for each category in the variable and all other components set to zero. Each category is represented as a separate feature in the resulting vector with a value of 0 or 1, depending on whether it was included in the initial data or not. Machine learning algorithms may effectively handle categorical variables and record their correlations with other variables by employing one-hot encoding [38]. Table 2 shows the features extracted from the dataset after pre-processing.

**Table 2:** Trauma dataset features

**Features**	**Categories**
Age	Child, teenager, young, middle-aged and elderly
Type of conveyance carrying to emergency	ambulance, taxi, personal vehicle
Place birth	city, village
Total expenditures	
Type of insurance	treatment services, social security, military, bank, free, others
Number of days admitted	One day, two days, three days and more
Sex	male, female
ICD-injuries	Pedestrian injured in transport accident, Pedal cycle rider injured in transport accident, Motorcycle rider injured in transport accident, Car occupant injured in transport accident, Water transport accidents, Slipping, tripping, stumbling and falls, Exposure to electric current, radiation and extreme ambient air temperature and pressure
Occupation	child, staff, worker, farmer, unemployed, students, businessmen, housewives, others job
ICD-external causes	Injuries to the head, Injuries to the abdomen, lower back, lumbar spine, pelvis and external genitals, Injuries to the shoulder and upper arm, Injuries to the elbow and forearm, Injuries to the wrist, hand and fingers, Injuries to the hip and thigh, Injuries to the knee and lower leg, Injuries to the ankle and foot
Education	child, illiterate, school, high school, after diploma
State of discharge	non-improvement, improvement

### Algorithm selection and evaluation

This section provides an overview of the association rule mining and Apriori algorithm.

#### Association rule mining

The goal of the data mining technique known as Association Rule Mining is to extract correlations, common patterns, or special structures from data repositories [39]. Association rules consist of two sets of items: the antecedent (or left-hand side) and the consequent (or right-hand side). These rules are typically expressed in the form X⇒Y, where X represents the antecedent and Y represents the consequent. The purpose of the analysis is to derive association rules that identify the items and cooccurrences of different items that appear frequently [40].

Strong association rules are those that meet the minimum support and minimum confidence thresholds as defined [41].

*Support*: The support of a rule indicates the frequency of its application in a given dataset [42].

1 $$\text{Support}\ ({\text{Item}}-{\text{A}}) =\frac{{\text{Frequnet}}({\text{Item}}-{\text{A}})}{{\text{N}}}$$

N: Total number of records

Definition of weight support in the present study:

2 $${\text{Support}}-{\text{Weight}}=\frac{({\text{Frequnet}}({\text{Item}}-{\text{set}})\times {\text{Harmonic}}-{\text{Mean}}({\text{Weight}}(\text{Element in Item}-{\text{Set}})))}{N}$$

*Confidence*: The confidence of a rule reflects the proportion of times that items in Y are found in transactions that also contain X [43].

3 $$\text{Confidence}\ ({\text{Item}}-{\text{A}},\mathrm{ Item}-{\text{B}}) =\frac{{\text{Frequnet}}({\text{Item}}-{\text{A}}\to {\text{Item}}-{\text{B}})}{{\text{Frequnet}}({\text{Item}}-{\text{A}})}$$

Definition of weight Confidence in the present study:

#### Apriori algorithm

The Apriori algorithm is a well-known approach for identifying frequent patterns in a dataset. These patterns consist of sets of items that occur frequently and exceed a predefined threshold known as the minimum support [44].

The Apriori algorithm consists of multiple phases or passes [41, 45]:

1. The first step involves generating candidate itemsets, where each k-itemset is created by combining (k-1)-itemsets that were identified in the previous iteration. A common pruning technique used in Apriori involves eliminating k-itemsets whose subsets, containing k-1 items, are not present in any frequent pattern of length k-1.

4 $$\text{Confidence}\ ({\text{Item}}-{\text{A}},\mathrm{ Item}-{\text{B}}) =\frac{{\text{Support}}-{\text{Weight}}({\text{Item}}-{\text{A}}\to {\text{Item}}-{\text{B}})}{{\text{Support}}-{\text{Weight}}({\text{Item}}-{\text{A}})}$$

2 The next phase of the Apriori algorithm involves calculating the support for each k-itemset candidate. This is accomplished by scanning the entire database to count the number of transactions that contain all the items in the k-itemset candidate. This step is a defining characteristic of the Apriori algorithm and requires scanning the entire database for the longest k-itemset.
3 To establish a high-frequency pattern, the Apriori algorithm identifies k-item sets that have a support greater than the minimum threshold. These high-frequency patterns consist of sets of k items.
4 If no new high-frequency patterns are identified, the Apriori algorithm terminates. Otherwise, the algorithm increments k by one and repeats the process from the first phase.

#### Evaluation

To compare the performance of the new proposed method and the classical algorithm, the following indices were calculated:

- RAM memory consumption
- The amount of space used on the hard disk
- Algorithm execution time
- Number of frequently generated patterns
- Number of generated rules
- Quality of generated rules: To calculate this, the median confidence value was calculated for different item sets in the new proposed method and the classical method.

#### Implemented framework

**Fig. 1:**
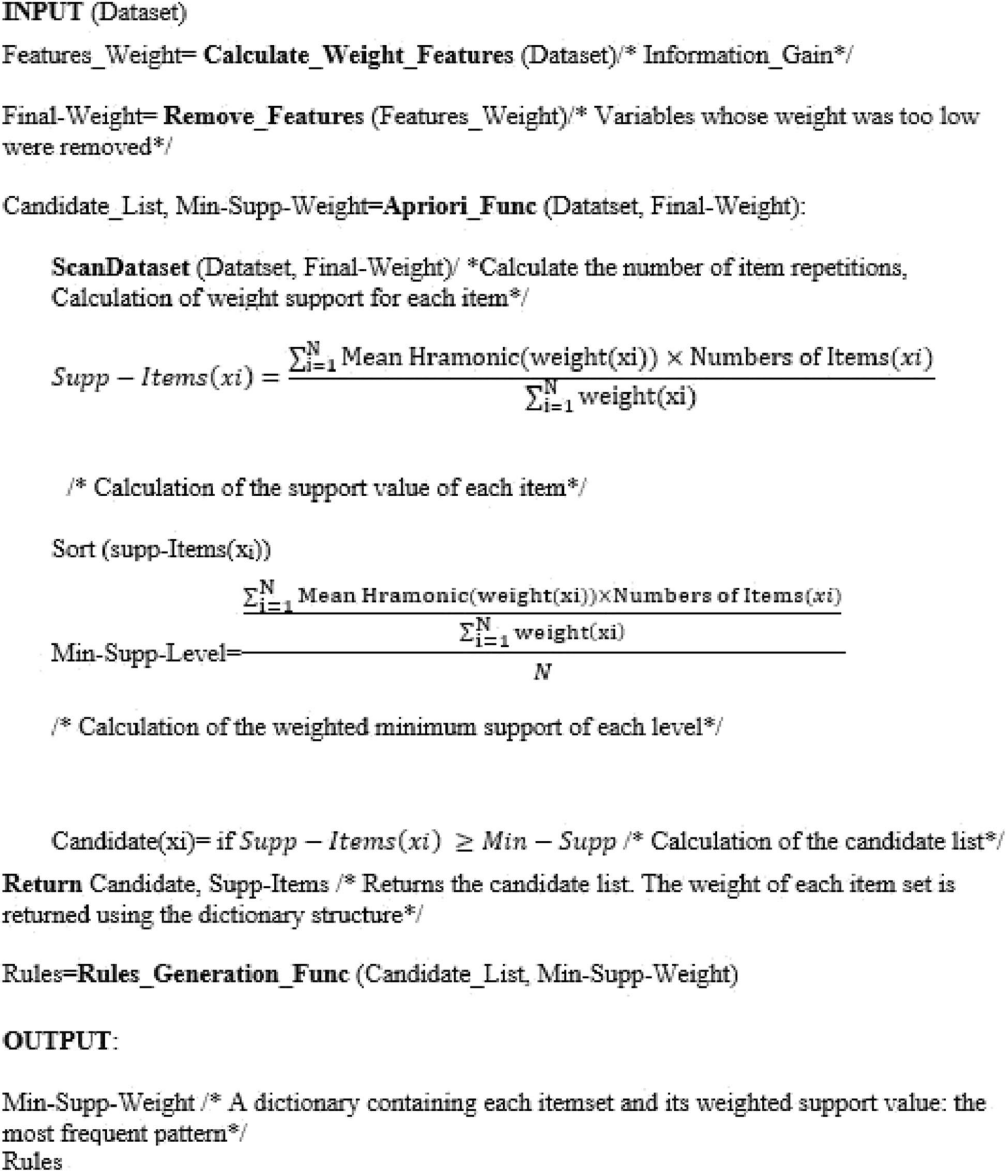
Pseudocode of the new algorithm

In this section, the new proposed algorithm is explained. This algorithm calculates its weight support based on the weight of each item and the number of repetitions of each item. Additionally, to determine the weight support of an item set, multiply the number of repetitions by the harmonic mean of the weights of its components. To determine the minimum weighted support at each level, divide the total weighted support of the item sets by the total records. Then, if the weighted support of each item set is greater than the minimum support of that level, that item set goes to the next stage as a candidate list. The pseudocode of the algorithm is shown in Fig 1.

1. The input of the algorithm is the dataset.
2. The weight value was calculated for all variables using the information gain method.
3. Variables whose weight was too low were removed.
4. The improved Apriori algorithm takes feature weights and dataset as input.
5. The support value of each item was calculated using the suggested formula:

5 $$Supp-Items\left(xi\right)=\frac{\sum_{{\text{i}}=1}^{{\text{N}}}\text{Mean Hramonic}\left({\text{weight}}\left({\text{xi}}\right)\right)\times \text{Numbers of Items}\left(xi\right)}{\sum_{{\text{i}}=1}^{{\text{N}}}{\text{weight}}\left({\text{xi}}\right)}$$

Supp-Items(xi): The supp-Items xi

Weight(xi): Information gain value for variable i

Mean Harmonic (Weight(xi)): The mean harmonic value of the i-th variable weight

Numbers of Items (xi): Number of itemsets xi

6 In this step, the amount of weighted support for each level was calculated using formula 6: the support for each item set was first calculated, then their sum was used to calculate the minimum support for that level.
7 Sort (Supp-Items(xi))

6 $$\text{Min-Supp-Level}=\frac{\frac{\sum_{{\text{i}}=1}^{{\text{N}}}\text{Mean Hramonic}({\text{weight}}\left({\text{xi}}\right))\times \text{Numbers of Items}(xi)}{\sum_{{\text{i}}=1}^{{\text{N}}}{\text{weight}}\left({\text{xi}}\right)}}{N}$$

N: Number of Record

8 According to formula 7, if the amount of support obtained for each item is greater than the minimum amount of support obtained for that level, that item set will go to the next stage as a candidate item set, otherwise it will be removed.

7 $$Supp-Items\left(xi\right) \ge Min-Supp$$

9 Then this process continues until no other item set is produced.
10 The Apriori improved algorithm function returns the most frequent patterns based on the weight of the variables and the minimum support value of each level.
11 Finally, the output of the algorithm is the frequent patterns and rules.

We examined the algorithm's essential elements to acquire a deeper understanding of its complexity.

- *Loading the Dataset (load Dataset)*: The process of reading an Excel file and transforming it into a matrix is characterized by a time complexity that scales with the size of the dataset. This can be represented as O(N), where N corresponds to the number of transactions or cells in the dataset.
- *Calculate Weights (information Gain)*: The time complexity associated with computing information gain for a feature can be denoted as O(N⋅V), where N represents the number of instances, and V stands for the number of unique values within the feature.
- *Remove Feature ()*: The time complexity for eliminating features with weights below 0.001 is typically O(n), where n represents the number of features in the data structure.
- *Creating Candidate 1-Itemsets*: The time complexity is O (N * M), with M representing the average number of items in a transaction.
- *Scanning the Dataset (scanD)*: The nested loops iterate through transactions and candidate itemsets, with a worst-case time complexity of O (N * M * K), where K denotes the length of the candidate itemsets.
- *Generating Candidate Itemsets (aprioriGen)*: The time complexity is contingent on the size of the existing frequent itemsets, with a worst-case scenario reaching O(2^(M-1)), where M represents the length of the frequent itemsets.
- *Calculating Primary Weights (primariweight)*: The time complexity is O (N * M), with M being the mean number of items in a transaction.
- *Apriori Algorithm Iterations (Apriori)*: The iterations entail examining the dataset and producing candidate itemsets. In the worst-case scenario, the time complexity is O (I * N * M * K), where I represents the number of iterations.
- Sorting (): The time complexity for sorting a list varies based on the sorting algorithm employed (e.g., Merge Sort: Time Complexity: O (n log n)).
- *Generating Association Rules (generate Rules)*: The time complexity is contingent on the quantity of frequent itemsets and their respective lengths. In the worst-case scenario, it can be expressed as O (F * (M^2)), where F represents the number of frequent itemsets, and M denotes their average length.

The Apriori algorithm's exponential nature results in significant computational costs, particularly when dealing with large datasets or when the minimum support threshold is set at a low value. In conclusion, the comprehensive time complexity of the Apriori algorithm is affected by various factors, including the dataset size (N), the average number of items per transaction (M), the length of candidate itemsets (K), the number of iterations (I), and the quantity of frequent itemsets (F).

## Experimental results

**Fig. 2:**
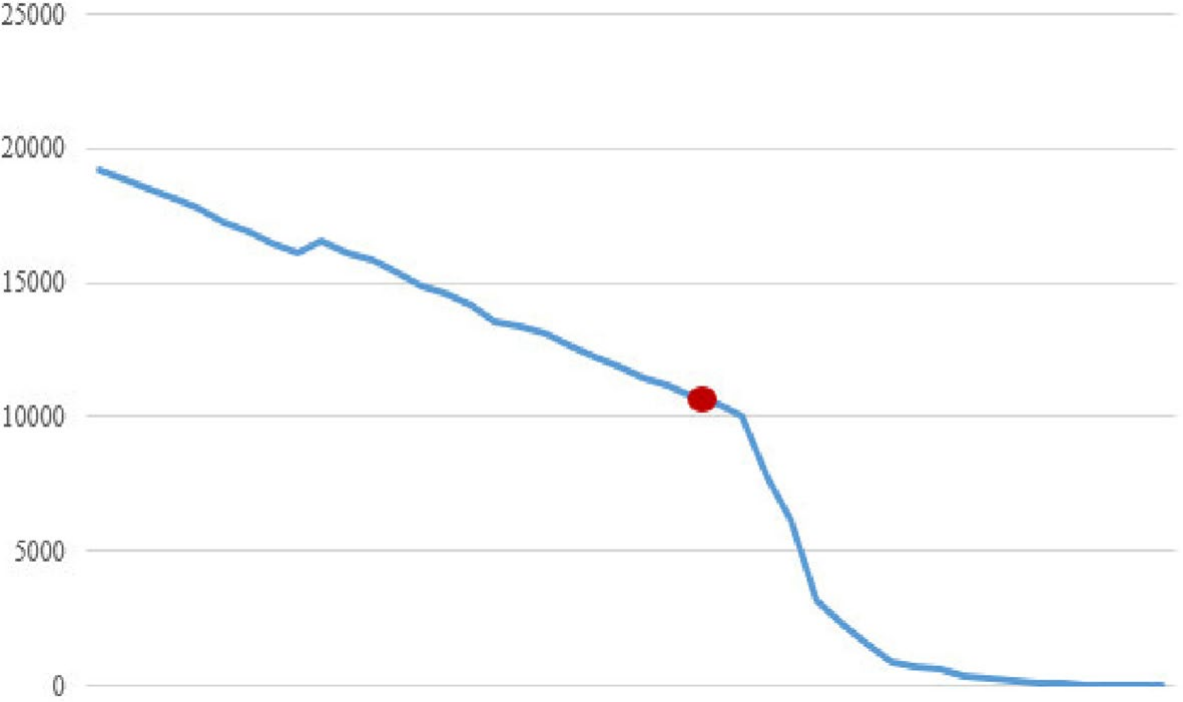
Number of frequent patterns

**Fig. 3:**
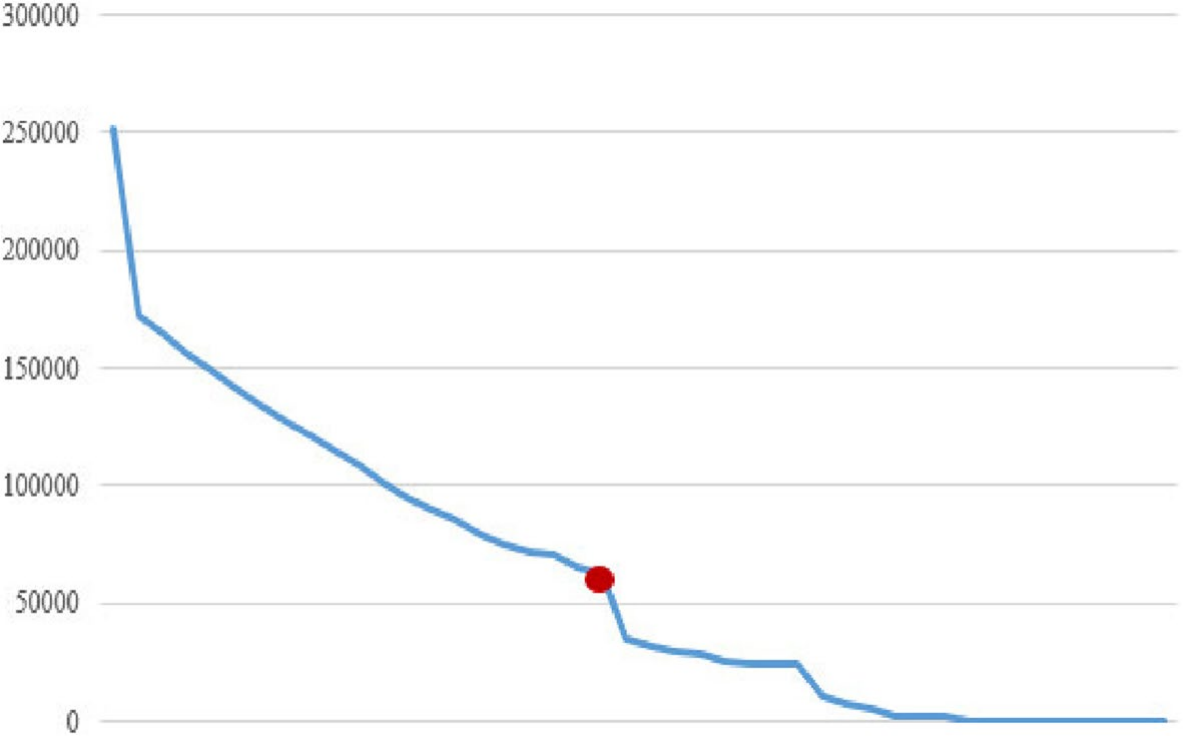
The amount of space used in the hard disk (KB)

**Fig. 4:**
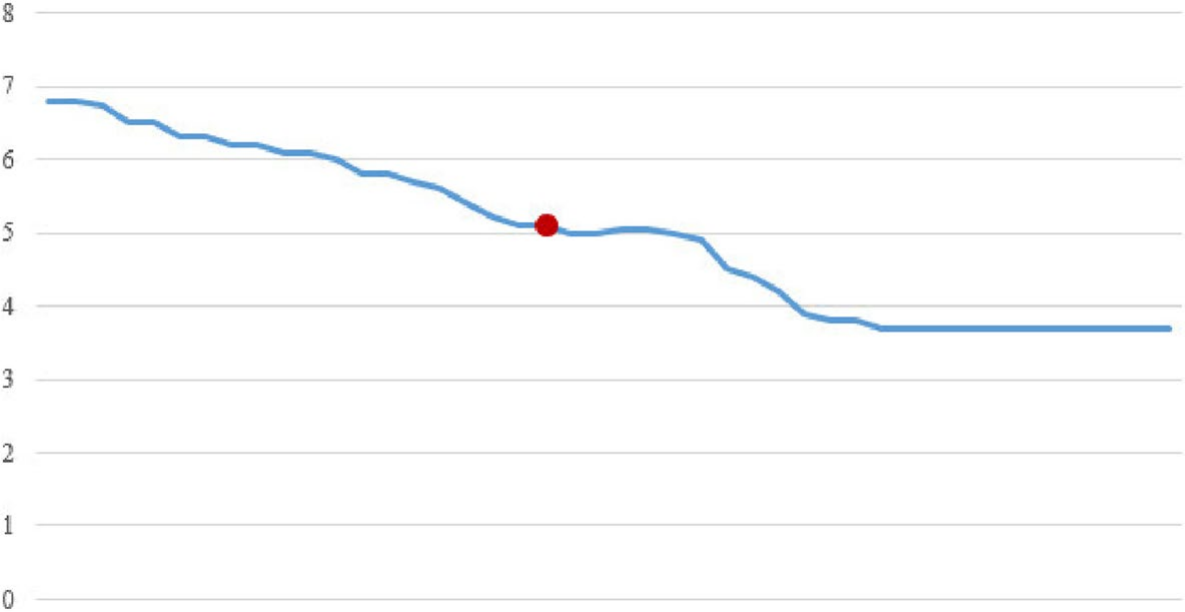
The amount of RAM memory (GB)

**Fig. 5:**
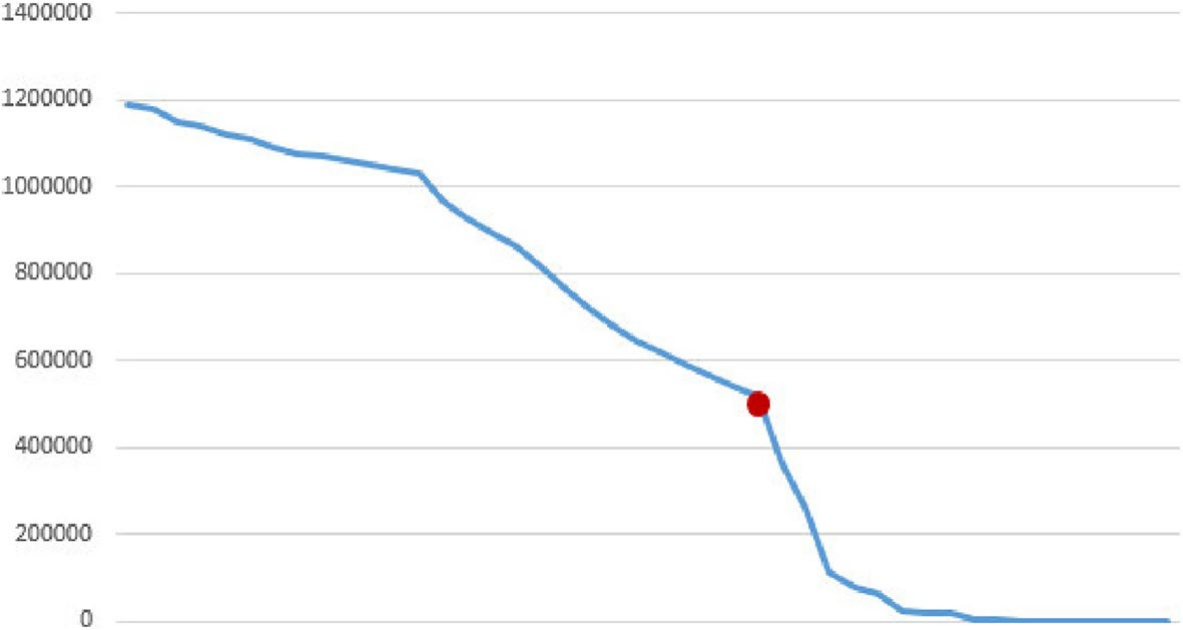
Number of generated rules

**Fig. 6:**
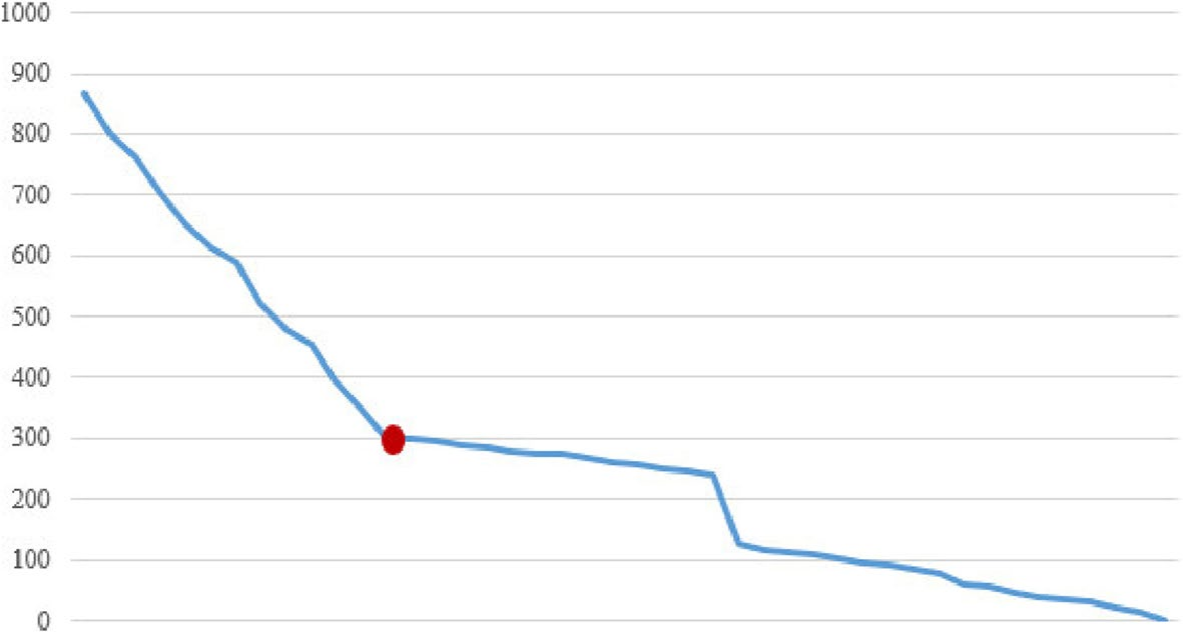
Duration of execution

**Fig. 7:**
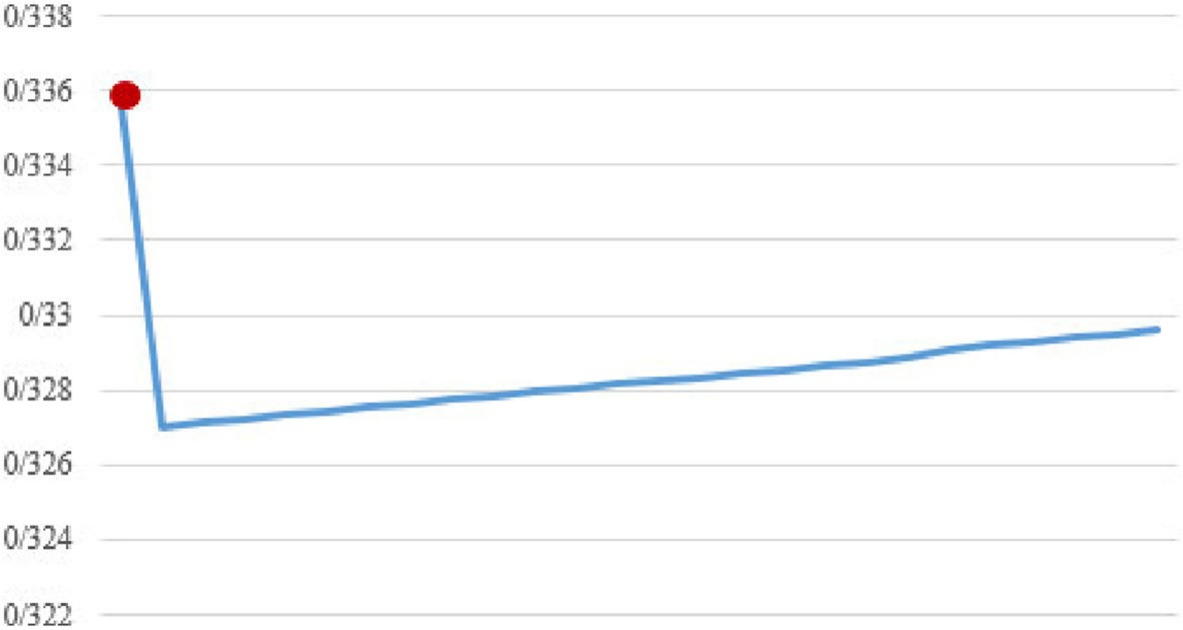
Confidence in four item sets (Median)

**Fig. 8:**
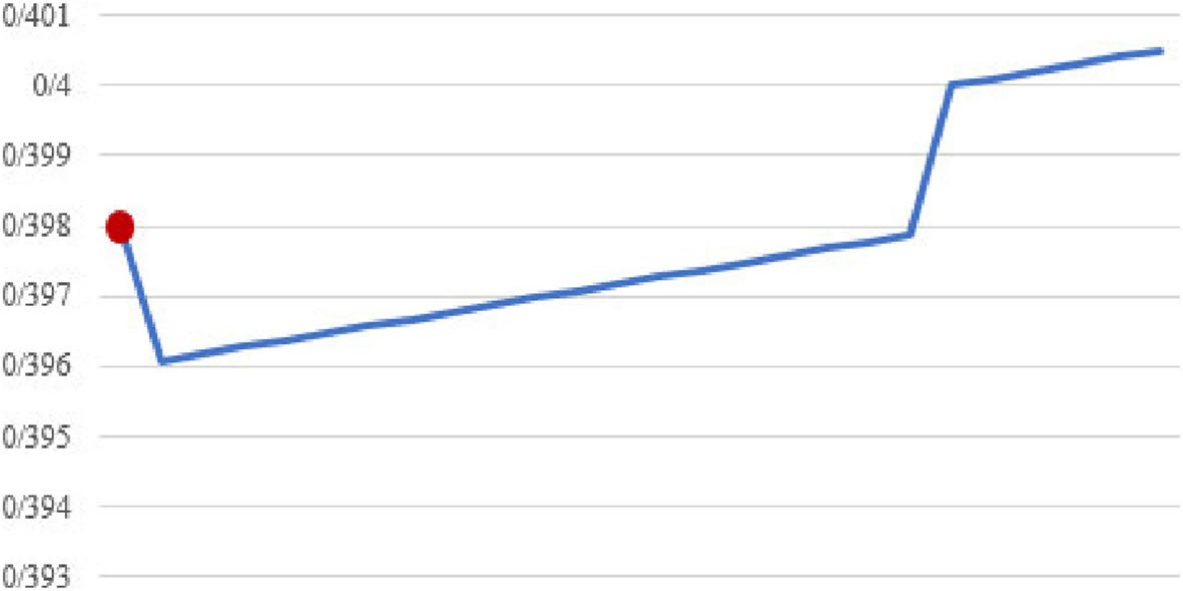
Confidence in seven item sets (Median)

**Fig. 9:**
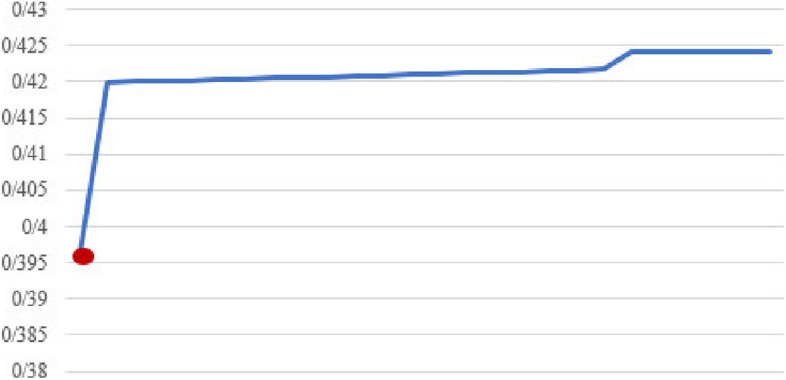
Confidence in nine item sets (Median)

Figures 2, 3, 4, 5, 6, 7, 8 and 9 show the performance of the proposed algorithm and the classical Apriori algorithm on trauma data. In Fig. 2, the number of frequent patterns generated based on the selection of different minimum supports in the classical algorithm of Apriori is shown. With the increase in the minimum support value, the number of frequently generated patterns has decreased. The red dot shows the number of frequent patterns generated using the new proposed algorithm.

In Fig. 2, the consumption of hard disk space from the generation of frequent patterns produced by the classical algorithm based on different minimum supports is shown. With the increase in minimum support, the consumption of hard disk space has decreased. The red dot in the diagram shows the amount of hard disk space consumed by the new algorithm.

Figures 4, 5, and 6 show the RAM memory consumption, the number of generated rules, and the execution time, respectively.

Figures 7, 8, and 9 show the median confidence values in four, seven, and nine item sets, respectively. In all these figures, the marked red dot shows the outputs of the proposed algorithm.

According to the findings, with the increase in the amount of minimum support, the number of generated patterns and rules has decreased, which reduces the amount of execution time and the amount of RAM and hard disk used.

Also, to evaluate the performance of the proposed method compared to the classical algorithm, the quality of the generated rules was also calculated. The quality of the rules in different item sets is very close to each other, and sometimes even in the new proposed method, the quality of the rules produced is better than the classical algorithm, according to Figs. 7, 8, and 9.

According to the findings of the research, one of the most frequent patterns with 80% confidence in the trauma dataset was:

Elderly patients who were hospitalized for more than 4 days had broken organs, and the cost of their treatment was also high and their insurance type was unclear. They were also taken to the hospital by motorcycle and experienced issues in the head and neck region.

It was also observed in some frequent patterns that patients were transported to the hospital by personal vehicle. Also, patients who did not improve experienced limb fractures.

Some of the most frequent patterns in the trauma data were:

Pedestrian injured in transport accident, Injuries to the head, personal vehicle, Elder, Expensive hospital fees.

Pedestrian injured in transport accident, Injuries to the hip and thigh, taxi, Elder.

Motorcycle rider injured in transport accident, Injuries to the shoulder and upper arm, taxi, worker, Cheap hospital fees.

Motorcycle rider injured in transport accident, Injuries to the head, personal vehicle, students, teenager.

Motorcycle rider injured in transport accident, Injuries to the head, Expensive hospital fees, Hospitalization for more than 4 days.

Pedestrian injured in transport accident, ambulance, One day of hospitalization, Cheap hospital fees, Injuries to the ankle and foot.

Pedestrian injured in transport accident, Injuries to the wrist, illiterate, teenager, Hospitalization for more than 3 days.

## Discussion

According to the findings of the research, the number of frequent patterns and rules produced in the new proposed method is much lower compared to the classic Apriori algorithm. The number of frequent patterns could be calculated for the minimum support greater than 0.045 in the classic Apriori algorithm, while for the minimum support less than 0.045, the calculation of the generated patterns is not cost-effective in terms of time, RAM memory, or even the amount of information generated. Therefore, it is not cost-effective to calculate all frequent patterns with the classic Apriori algorithm. Also, due to the fact that the generated rules are made from frequent patterns and their number will be several times that of the frequent patterns, in the classic Apriori algorithm, calculating all the generated rules will not be cost-effective.

The importance of variables and the production of minimum support based on level by level in the new method cause the minimum support to be different for different levels, while in the classic method, one value is considered the minimum support for all levels. But in the new proposed method, one value is generated for single itemsets, one value for binary itemsets, and different amounts of minimum support are generated for other itemsets.

Also, the minimum support in the new proposed method automatically weights the variables and calculates the minimum support at all levels without user intervention. This makes it easy to calculate the generation of frequent patterns and the resulting rules based on the importance of variables in the case of datasets that are unknown to the user.

In [21] a data mining technique was applied in a novel way to identify IBIPs that were linked to co-occurring injury categories. This analysis demonstrated significant differences in IBIPs between various types of road users, which can provide valuable insights into how injury severity and outcomes may vary. These findings could have important implications for prioritizing crash countermeasures. In [22] determined that Data mining through association rule mining could potentially be the optimal method for determining the key factors that impact mortality rates in trauma patients with hospital-acquired infections. Among these factors, advanced age, tracheal intubation, mechanical ventilation, surgical site infections, skin infections, and upper respiratory infections appear to be the most crucial risk factors that contribute to mortality rates. In [23], Their approach differed from previous studies that examined the correlation between high levels of life trauma, depression, and suicide using statistical analysis. Instead, they utilized a distinct methodology that identified highly correlated patterns and effects between trauma, depression, and suicide in primary school students. Through the use of FP-Growth association rule mining, this study was able to determine the linked patterns between high-life trauma, depression, and suicide among primary school students attending extended opportunity schools in rural Thailand. The findings revealed a total of 34 associated patterns for high trauma, 14 associated patterns for depression, and 35 associated patterns for suicide. In [25] has demonstrated how association rule mining can be used to extract sets of rules between different diagnosis types and laboratory diagnostic test requirements. Real-life data from emergency departments of a large-scale urban hospital were utilized in the research.

Despite the use of the Apriori algorithm in these studies, they have benefited from its classical type. In studies [21–25] a classical Apriori algorithm with manual input of minimum support has been used to extract frequent patterns.

In studies [27, 35, 36] despite the improvement of the classical Apriori algorithm with multiple selections of the minimum support, the minimum support value was still entered by the user, and the variable weights were not considered. Although the calculation of minimum support is automatic in studies [32, 46], the variable weights were not taken into account. Despite using the weight to determine the association rules in studies [33] and [34], the user enters the minimal support value manually. However, in this study, Variable weighting and automated minimum support determination are employed to generate frequent patterns.

In studies 1, 2, and 3, multiple minimum supports were employed to modify the minimum support threshold. However, a notable limitation of the aforementioned approach is the reliance on user intervention for determining the minimum support. The challenge of automatically generating the minimum support has not been addressed in these three studies, although they represent an improvement compared to the classic Apriori method. Additionally, while real dataset was utilized in these studies, the clarity of algorithm implementation remained unclear. Evaluation metrics in study 1 included the Number of Large Item Sets and Number of Candidate Item Sets. Study 2 focused on Runtime, while study 3 utilized the Number of Frequent Patterns for evaluation.

In study 4, while the authors present a novel approach to efficiently retrieve the top few maximal frequent patterns in order of significance, eliminating the need for the minimum support parameter, they still require users to specify another parameter, namely the desired number of itemsets denoted as “k”. This signifies a form of user intervention in the algorithm.

In study 5, the research strives to introduce an innovative algorithm for association rule mining with the aim of improving computational efficiency and automating the identification of suitable threshold values. The proposed method utilizes the particle swarm optimization algorithm, which initially pursues the optimal fitness value for each particle. However, one of the limitations acknowledged by the study's authors is the absence of consideration for variable weights. The evaluation platform employed was Borland C++ Builder 6, and the assessment criteria included Runtime and the Number of Frequently Generated Patterns.

In studies 6 and 7, despite notable advancements in the automated calculation of the support threshold and the utilization of various statistical indicators for this purpose, there is a notable omission in considering the weight of the variables. The specific platform employed for their study was not disclosed, and their evaluation criteria encompassed the Number of Association Rules, Time Consumption, and the Quality of the Extracted Rules.

In the recent study, a novel approach was introduced that focuses on variable weighting and utilizes the harmonic mean for the automatic calculation of the minimum support. This method was implemented using Python. Unlike previous studies, this approach considers the weight assigned to variables, acknowledging its significance in the mining process.

The evaluation process in this study was comprehensive, involving various indicators to assess the performance of the proposed method. These indicators encompassed RAM memory consumption, the amount of space utilized on the hard disk, algorithm execution time, the number of frequently generated patterns, the number of generated rules, and the quality of the generated rules. Such a multi-faceted evaluation provides a more holistic understanding of the method's effectiveness across different dimensions, addressing not only computational efficiency but also the quality of the extracted patterns and rules.

In the present study, similar to other investigations, association rule mining successfully identified frequent patterns within the data obtained from Kashan Trauma Centre. These patterns have the potential to significantly enhance healthcare outcomes. For instance, it was observed that the majority of patients who did not improve had limb fractures. Consequently, the healthcare team can prioritize their attention towards patients with fractures. Additionally, it was noted that the patients involved in such incidents were predominantly motorcycle riders. Hence, there is an opportunity to raise awareness among the general public regarding the hazards associated with motorcycle usage, and it is advisable to promote the use of appropriate safety gear while riding motorcycles. Furthermore, in some cases, these patients were transported to the hospital in personal vehicles. Thus, it is essential to educate the general public about the importance of adhering to safety precautions when transporting patients in personal vehicles. In some of the produced patterns, the elderly was at risk, so it is suggested to teach their families to take care of the elderly.

Among the limitations of the research was that the duration of the algorithm execution and the amount of memory consumption depend on the hardware device. In this research, a device with 48 GB of RAM, Core i5 generation 9 was used, which was expensive for the researchers. Also, in this study, we were looking for the automation of min support rather than the optimization of time and memory consumption. However, we were able to reduce time and memory compared to the classical Apriori algorithm.

## Conclusion

Association rule mining shows potential as a tool for applications in trauma research and treatment. In the examination of trauma registries and large healthcare datasets, its capacity to detect frequent item sets and association rules is particularly relevant and yields insightful findings and knowledge. It can play a crucial role in upgrading trauma treatment systems, detecting risk factors, and forming preventive strategies by looking for associations and trends within trauma-related data. Its incorporation into the healthcare industry could improve decision-making procedures, create efficient regulations, and improve patient outcomes. Integrating association rule mining into trauma research offers a chance to advance trauma therapy and ultimately enhance patient wellbeing as data mining continues to develop. While the generation of frequent patterns in large datasets based on the classic Apriori algorithm and selecting the minimum support manually is not cost-effective in terms of time and memory consumption, calculating the minimum support based on the weight of variables and different levels of item sets can improve the classical algorithm and be used in various industries, including the health industry and large datasets such as trauma, to extract frequent patterns.

## Data Availability

All data generated and analyzed during the current study are not available to the public but may be obtained from the corresponding author upon reasonable request and with permission from the Kashan University of Medical Sciences.
